# Relative Age Effect in UEFA Championship Soccer Players

**DOI:** 10.1515/hukin-2015-0079

**Published:** 2015-10-14

**Authors:** Sixto González-Víllora, Juan C. Pastor-Vicedo, David Cordente

**Affiliations:** 1 Faculty of Teaching Education (Cuenca), University of Castilla-la Mancha, Spain. EDAF research group.; 2 Faculty of Teaching Education (Toledo), University of Castilla-la Mancha, Spain. EDAF research group.; 3 University of Castilla-la Mancha, Spain.

**Keywords:** relative age effect, amateur vs. professional players, international soccer, talent identification

## Abstract

Relative Age Effect (RAE) is the breakdown by both age grouping and dates of birth of athletes. In the past 20 years the existence of this effect has been shown with higher or smaller impact in multiple sports, including soccer. The purpose of this study was to identify the existence of RAE in European soccer players. The sample included 841 elite soccer players who were participants in the UEFA European Soccer Championship in different categories. The professional category (n = 368), U-19 (n = 144) and U-17 (n = 145) were in 2012, and U-21 was in 2011 (n = 184). The Kolmogorov-Smirnov test and the Levene test recommended the use of nonparametric statistics. The results obtained by the square test ( 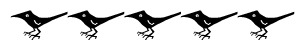 the Kruskal-Wallis test and Cohen’s effect sizes revealed the existence of RAE (χ^2^ = 17.829, p < 0.001; d = 0.30), with the size of their different effects depending on their category or qualifying round achieved by the national team and the existence of significance in the observed differences by category. Therefore, we could continue examining RAE which is present in elite soccer, and could be considered a factor that influences performance of the national teams tested. RAE was not evident in the professional teams analysed, however it was present in the three lower categories analysed (youth categories), with its influence being greater on younger age categories (U-17).

## Introduction

The Relative Age Effect (RAE) refers to ‘asymmetry in the birth-date distribution favouring players born early in the selection year and discriminating against participants born later in the year’ (Helsen et al., 2012). RAE has been studied in a large number of sports. Due to the number and quality of the studies, there are two sports which stand out among others: soccer and ice hockey — the last one being the first in this research field of sport (Grondin et al., 1984). Grondin et al. (1984) were the first authors who considered a possible relationship between the month of birth and the sports performance, having analysed volleyball and ice hockey teams at a recreational, competitive and senior level during the 1981–1982 season.

The term ‘relative age’ depends on the date of birth related to the selection data used to place a child in a specific age group (Wattie et al., 2008). The RAE is strikingly evident in activities that are competitive and where performance is highly correlated with age and the level of maturity (Thompson et al., 2004). In European countries, the majority of team sports, soccer among them, are made up of participants born between the 1st of January and the 31st of December of the same year, but occasionally spanning two consecutive years. Thus, ‘a child born at the beginning of a given year will be almost 12 months older than another athlete born at the end of the same year. Nevertheless, they will compete together’ (Gil et al., 2014).

In all the categories it was observed that there was a higher percentage of players born in the first quarter of the year. With the objective of making competitions fairer, different sport organisations take into account models of classification based on the players’ date of birth (Helsen et al., 2005). However, a competitor born in the first quarter of the year may show a significant difference in maturation compared to another player born in the last quarter of the same year, in spite of belonging to the same sports category (Baxter-Jones, 1995). Baker et al. (2010) note that it is not fully explained how the maturation advantages in the early ages produce sporting achievements in the long term.

The maturing process has been demonstrated, in terms of physical differences, to have the biggest effect at the beginning of puberty (La Rochebrochard, 2000). This situation is not only present in the sports (Gil et al., 2014; González-Víllora and Pastor-Vicedo, 2012; Helsen et al., 2012); it can also be found within the educational environment in terms of academic performance (Bedard and Dhuey, 2006). Consequently, the problem of RAE is something global that implicates different environments; it is not unique to sports.

Since it has been confirmed that RAE exists in sports (Dudink, 1994), and particularly in soccer (Gil et al., 2014; González-Víllora and Pastor-Vicedo, 2012; Helsen et al., 2005), some authors have focussed on observing what characterised those players born in the first quarter of the year. For example, within the entire group of young male and female participants of the French Federation of Basketball, it has been noted that players born in the first quarter of the year are more developed in body height, mass and strength (Delorme and Raspaud, 2009). These advantages are reflected in their potential, contributing to their identification by sports experts (Helsen et al., 2005). This fact promotes earlier incorporation in sports schools, which will accelerate the development of technical and tactical (Ward and Williams, 2003), physical, physiological abilities, as well as anthropometric characteristics and conditional capacities (Delorme and Raspaud, 2009; Unnithana et al., 2012) along with psychological skills as self-regulation of attention, emotion and memory (Martin et al., 2004).

An advantage has been provided to senior players in the selection process of sports talent. This hypothesis on the chances of being selected in soccer according to anthropometric and physiological characteristics was already suggested by Reilly et al. (2000). The selection of players is of utmost importance as sometimes it carries the possibility of training players with a higher skill and experience level, as well as being trained by qualified and experienced professionals (Sherar et al., 2007). In sports like soccer, this process either promotes or precludes the possibility of being part of a team A, within each training category, so there are soccer schools’ teams in the same category (team A, team B, team C, etc.). If one player can play in team A, which conditions the possibility of playing in certain competitions and tournaments that favour playing with more competitive teams (Helsen et al., 2005), it determines positively the development of the player. This fact is developed within each club, but it is also produced in the process of selection for regional and national teams at youth levels, and even in the early years of a senior level.

RAE has been identified in baseball (Stanaway and Hines, 1995), ice hockey (Weir et al., 2010) and tennis (Baxter-Jones et al., 1995). By contrast, in other sports such as soccer (Stanaway and Hines, 1995) and gymnastics (Baxter-Jones et al., 1995), RAE has not been properly appreciated. More recently, RAE has been clearly identified in soccer (Delorme et al., 2010; Gutiérrez-Díaz et al., 2010; Helsen et al., 2005). It has been studied in national league players, including both professional and amateur young players, from Germany (Augste and Lames, 2011; Musch and Hay, 1999), Australia, Brazil, Japan (Musch and Hay, 1999), Belgium (Helsen et al., 2000), Spain (Gutiérrez-Díaz et al., 2010), France (Delorme et al., 2010) and the United Kingdom (Dudink, 1994). To exemplify one of them, Augste and Lames (2011) not only found evidence of RAE in the U-17 German elite soccer, but were also able to relate the potential success of a sports team with the date of birth of their players, which means that there is a greater probability of finishing closer to the top of the league rankings when RAE is higher. Compiling the results of RAE in soccer, it is demonstrated that professional players born in the first half of the year represent about 60% of the total number of players calculated (Musch and Hay, 1999). If we focus on international championships with players selected by their respective countries, there are fewer studies on RAE. In the first study, Barnsley et al. (1992) observed that in the 1990 Soccer World Cup, 55% of the players were born in the first half of the year. This figure increased to an average of 79% in international U-17 and U-20 competitions. Another representative study is the research done with young international players by Helsen et al. (2005), which analysed 2,175 subjects. This study focused on analysing the national U-21, U-18, U-17, U-16 and U-15 teams in the 1999–2000 season. RAE results show significant differences in the selections of Denmark, Belgium, England, France, Germany, Italy, the Netherlands, Spain and Sweden. The greatest disparity among those born in the first quarter of the year and the last was found in the selections of Germany (50.49% in the first quarter against 3.89% in the last) and England (50% and 17%, respectively). There is no evidence of RAE in the national teams of Portugal or in the U-21 male and female U-18 (the only one analysed in this genre).

The purpose of the present study was to examine the dates of birth of the international players, together with other variables in the 2012 European Soccer Championship at a senior level and in U-21, U-19 and U-17 from the previous European Soccer Championship. The proposed hypothesis was that players born in the first quarter of the year would be over-represented compared to those born in later quarters of the same year, with RAE being more significant in categories where players were younger, although having a smaller effect on professional players.

## Material and Methods

### Participants

In the present study, a total of 841 male players participating for their country in different categories in the European Soccer Championship were considered. The analyzed players, from 16 teams (Spain, Italy, Ireland, Croatia, Poland, Greece, Russia, Czech Republic, Holland, Denmark, Germany, Portugal, Ukraine, Sweden, France and England) participated in the Absolute Football Eurocup of 2012 where the final tournament was hosted by Poland and Ukraine (n = 368). All of them played three matches except teams who played quarter-finals (Spain vs. France; Germany vs. Greece; Italy vs. England; Czech Republic vs. Portugal) as they played four matches, the teams who achieved semi-finals (Spain vs. Portugal; Italy vs. Germany) who played five matches, as well as the teams who played the final (Spain vs. Italy) who played a total of six matches. The winner was Spain, who participated with 23 players with body height of 180.4 cm and a birth date average of the 17^th^ of May of 1985.

For the U-21 category held in 2011 in Denmark (n = 184), the eight teams who achieved the qualification to the final tournament were selected (Spain, Czech Republic, Denmark, Ukraine, England, Iceland, Belarus and Switzerland). All of them played three matches except teams who achieved semi-finals (Switzerland vs. Czech Republic), Olympic playoff (Czech Republic vs. Belarus) or the final (Spain vs. Switzerland), who played five matches each. The winner was Spain, who participated with 23 players with body height of 181.0 cm and a birth date average of the 18^th^ of May of 1989.

For the U-19 category hosted by Lithuania in 2012 (n = 144), eight teams were selected (Spain, Greece, Portugal, Estonia, England, France, Croatia and Serbia). All of them played three matches except the teams who played semi-finals (Spain vs. France; England vs. Greece) with four matches, or teams who achieved the final (Spain vs. Greece) with five matches. The winner was Spain, who participated with 18 players with body height of 179.4 cm and a birth date average of the 15^th^ of June of 1994.

With regard to the U-17 held in 2012 in Slovenia (n = 145), the best eight teams who achieved the final stage were selected (Spain, Poland, Holland, Germany, France, Slovenia, Georgia and Iceland). All of them played three matches except the national teams who played the semi-finals (Germany vs. Poland; the Netherlands vs. Georgia) with four matches, or the teams who arrived to the final (Germany vs. the Netherlands), who played five matches. The winner was the Netherlands, who participated with 18 players with body height of 180.3 cm and a birth date average of the 14^th^ of May of 1995.

### Measures

The statistical data in this article was taken directly from the official website of the Union of European Football Associations (UEFA), available at www.uefa.com. This website provides the statistical data regarding the player’s name, day, month and year of birth, as well as the country the player represents, the playing position, the category in which the player competes, the year in which the competition was played and the result achieved. For all categories analysed, only the teams that had participated in final stages of competitions were considered. This led to obtaining information from 16 selections in the senior level and eight selections in each of the categories following U-21, U-19 and U-17.

### Analysis

The cut-off date for the soccer competition year is the 1^st^ of January. As such, January was selected as the first month of the selection year and December as the last. The birth month of each player was compiled to define the birth quarter (Q), and four birth quartiles were designated (Q1 = January to March; Q2 = April to June; Q3 = July to September; Q4 = October to December). Relative age was also coded into half year categories (i.e., S1 and S2).

The Kolmogorov-Smirnov test (*p* < 0.05) and the Levene test (*p* < 0.05) were used to reject the normal distribution of the variables studied, suggesting the use of nonparametric statistics. The Chi-square test ( 
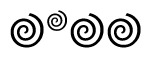
 was used to compare the relative age quartiles for each category based on the age group and the teams that reached the semifinals, finals and the championship (team results). Subsequently, differences between the observed and expected birth date distributions were analysed. Chi-square statistics did not reveal the magnitude and direction of an existing relationship. Significant chi-square values were, therefore, followed up by calculating Cohen’s effect size for the half-year distribution (S1 vs. S2) in order to examine subgroup differences by category and team results, with respect to the bias of the birthdate distribution. Cohen’s effect sizes (*d*) were used for interpretation of small (0.20), medium (0.50) and large (0.80) effects. Furthermore, differences between categories were calculated using the Kruskal-Wallis test. All analyses were carried out using SPSS 19.0, and statistical significance was set at *p* < 0.05.

## Results

**Figure 1:**
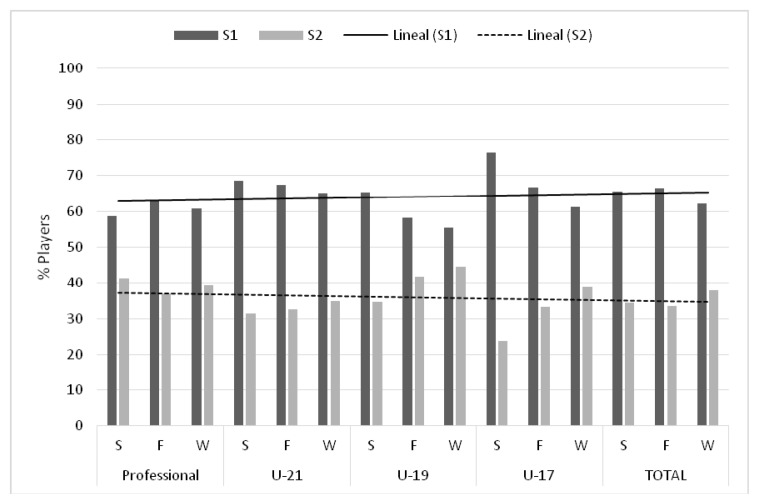
Distribution of soccer players per semester differentiated by category

Table 1 shows the players who participated in the different categories of the Eurocup, distributed by the quartile of birth. It should be noted that all categories showed a different distribution depending on the quartile in which players were born. However, only in the categories U-21, U-19 and U-17 the distribution of players was significantly different. This result was not enough to say that the observed differences were due to the existence of RAE. To identify this effect we calculated the effect size (d) comparing subjects from S1 to S2. With the intention to supplement the information showed in Table 1, Figure 1 was drawn, showing the distribution of players per semester, differentiated by category. This calculation reflected that RAE was present in the youth categories (U-21, U-19, U-17), with an average effect in the categories U-21 and U-19, and a higher effect in the U-17 category. Moreover, the Kruscal-Wallis test revealed the existence of significant differences among the four categories studied (χ^2^ = 17.829, *p* < 0.001).

In order to further deepen the study of RAE, it was also appropriate to evaluate the existence of this effect in the final stages of the competitions analysed and its possible influence on the results obtained from teams participating in the Eurocup. In this sense, Table 2 describes the distribution of each player according to the category to which he belongs, the quartile in which he was born and the result obtained by his selection, identifying it by the playoffs reached. It should be noted that the professional category is the only one that has a quarter-end in its final playoff; the rest of playoffs (semifinal, final) are identical. The winner variable shows the number and the percentage of players per quarter who played in the winning team of the tournament.

Observing data in Table 2, it can be seen how the uneven distribution of players according to the quartile of birth persists within each playoff and in the final result. However, this distribution is significant only in the semifinals of the categories U-21 and U-17, without any significance being found in the rest of the categories.

**Figure 2:**
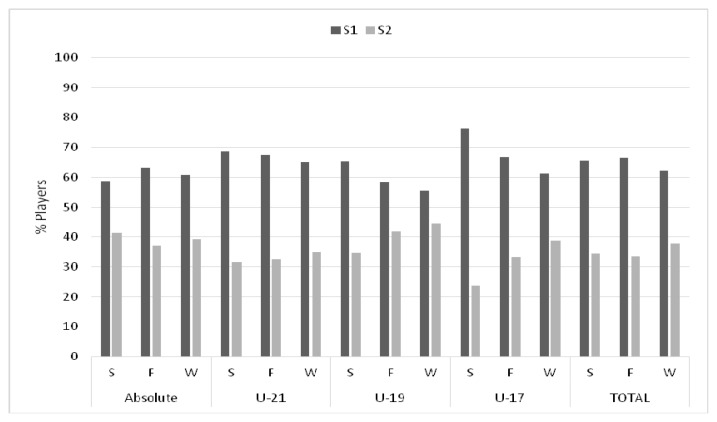
Distribution of soccer players per semester differentiated by category and team results

**Figure 3:**
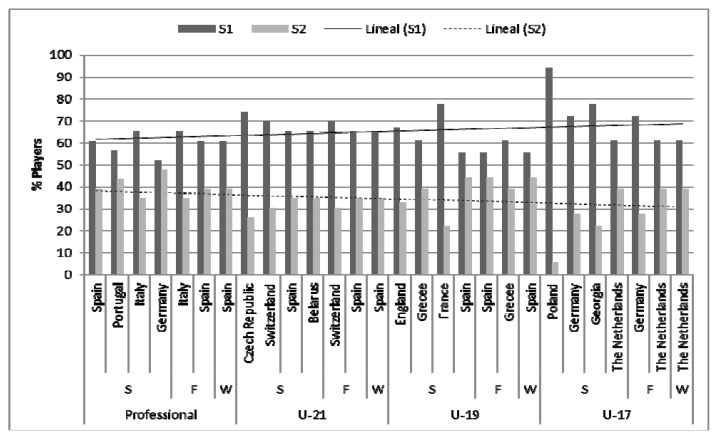
Distribution of soccer players per semester differentiated by selection and qualifying round reached S: Semifinal; F: Final; W: Winner.

The most interesting aspect is reflected in the calculation of effect size. Consequently, with the intention to supplement data showed in Table 2, Figures 2 and 3 were drawn. It is thus evident that there is still a clear preference for players born in the first six months of the year, and that this preference is greater as the category diminishes, as reflected in Figure 2 and its trend lines (S1 and S2). However, according to the results reported in Table 2, it should be noted that in the professional category no RAE is observed, whereas in the U-19 category RAE is observed in the final match with a high effect. In this sense, the presence of RAE in the categories U-21 and U-17 is more evident. In fact, it can be seen how the presence of this effect is significant in both qualifying playoffs considered, being high in the semifinals and average in the final match. In addition, it can also be observed how this effect size appears to be much higher in the semifinal of the U-17 category compared to the U-21 category.

On the other hand, in order to examine the persistency of RAE in this kind of competition where all the soccer players are selected from the top of their categories and countries, Figure 3 is presented, where all players are shown, differentiated by category, semester and the final result reached by their respective national teams. Thereby, it can be observed in Table 2 how the distribution of players per quartiles is significantly different in the semifinals and the final, but also within the overall winners of the tournament. Comparing the distribution of players in S1 and S2 (Figure 2 and 3), it can be noted that RAE is present and significant, with an average size in the selections playing the semifinals and final, as well as among teams who won each international tournament, increasing the size of the effect from the semifinal to the final game (Figure 3).

## Discussion

The present study analysed the influence of RAE within each group of players in the Football Eurocup: Elite, U-21, U-19 and U-17. This effect was assessed by the level of performance reached in each competition, categorising the teams that had not passed from the qualifying group and those who reached the semifinal and final games. Therefore, we should emphasise the quality and range of the study sample, as there is no literature about RAE in soccer and international competitions which analyses four different categories. At the same time, it is observed that RAE is present in professional soccer and seems to have some influence on the final result of the competition as all teams reaching the final stages in the tournament are those having a bigger RAE among its players; thus, RAE is related to athletic performance. Having observed the analysis made of the semifinals and final, it can be stated that selections with a greater effect of RAE are those which more often reach the final stages of the tournament. RAE may be one of the factors that determine performance of international teams in soccer.

Soccer has been one of the most studied sports with regard to RAE, although RAE in soccer has never before been related to performance in the UEFA European Soccer Championship in different categories. As it was noted in the introduction, this phenomenon had been analysed to a greater extent in national leagues, with no studies focusing on international competitions. This is somewhat unusual because players participating in these championships are selected and represent best players in their countries. The first study evaluating RAE in national teams was written by Barnsley et al. (1992), who found in the 1990 World Cup that 55% of players were born in the first half of the year. In the U-17 and U-20 World Cup, a greater bias was shown, as in the average of both, 79% of players were found to have been born in the first half of the year. Helsen et al. (2005) also analysed the ranks of the national teams (U-21, U-18, U-17, U-16 and U-15) in the 1999–2000 season. The national teams of Germany (50.49% in the first quarter, 3.89% in the fourth quarter) and England (50% and 17%, respectively) were the ones with the greatest impact of RAE. RAE was not observed in Portugal in the U-21 male category. If these data are compared to those presented in the results section of the present study, it could be noted that RAE has been reduced over the years. This is a positive development, although RAE still remains today.

Helsen et al. (2012) compared the RAE of professional soccer players in 10 European countries over a 10-year period (2000–2001 and 2010–2011 competitive seasons). Generally, results indicated no change in the RAE over the past 10 years in professional soccer (Chi-square goodness-of-fit tests). Despite these results, Helsen et al. (2012) proposed a change in the structure of youth involvement to reduce the impact of RAE on the soccer talent identification process.

In recent years, several hypotheses have been considered to explain the existence of RAE (González-Víllora and Pastor-Vicedo, 2012). One of the most widespread and justified on a scientific level has been the maturational hypothesis (Delorme and Raspaud, 2009; Delorme et al., 2010; Deprez et al., 2013; Helsen et al., 2000; Reilly et al., 2000). This hypothesis is supported by the potential maturational differences due to the difference in chronological age in children born within the same year (González-Víllora and Pastor-Vicedo, 2012). When sports teams select young players, there is a negative bias towards players born at the end of the year, since they are less mature and have anthropometric, physical and cognitive disadvantages (Delorme and Raspaud, 2009; Unnithana et al., 2012). According to Gutiérrez-Díaz et al. (2010), it is more likely that RAE is greater when the clubs have more reputation and financial resources, as they have a greater opportunity to select players. Therefore, this bias could lead to a systematic discrimination in the professional recruitment of players (Delorme et al., 2010).

Roberts et al. (2012) analysed the data of physical cardiorespiratory condition (11,404 children aged 9–10 years and 3,911 children aged 11–12 years) in relation to the month of birth. The results showed a significant relationship between the month of birth and the measured parameters (p < 0.01), even when controlling somatic maturation (p < 0.05). Deprez et al. (2013) analysed the anaerobic performance characteristics and the anthropometrical characteristics in Belgian elite youth soccer players (374 individual soccer players divided into three age categories: U-13, U-15 and U-17). It was shown that in anthropometric variables there were no significant differences except for body height in the U-15 age group.

Gil et al. (2014) evaluated the relationship between RAE and anthropometry, maturity and performance in youth soccer players (88 youth soccer players with age 9.75 ± 0.30). Anthropometric measurements, physical tests (sprint, agility, endurance test, jump and hand dynamometry) and estimation of the maturity status were carried out. Older players were taller (p < 0.05), had longer legs (p < 0.01) and a larger fat-free mass (p < 0.05). Maturity offset was smaller in the older boys (p < 0.05); however, age at peak height velocity was similar. Older boys performed better in velocity and agility (p < 0.05) and particularly in the overall score of performance (p < 0.01). Stepwise regression analysis revealed that chronological age was the most important variable in the agility test and the overall score, after the skinfolds (negative effect). Gil et al. (2014) concluded that these differences may underlie the RAE.

However, maturity does not refer only to the physical condition, but also to mental development including the self-control of attention and emotions, as well as other functions such as memory (Martin et al., 2004). Some research suggests that there is a predisposition for being selected in soccer when taking into account anthropometric and physiological characteristics (Reilly et al., 2000) or the development of cognitive and perceptual abilities (Ward and Williams, 2003). Thus, relatively younger players can offset RAE if they enter into puberty earlier (Deprez et al., 2013).

There are other hypotheses that attempt to explain the phenomenon of RAE, such as previous experience (Musch and Grondin, 2001). Players born earlier in the year have more opportunities to play and practise and have better access to training and competition, and so are better able to develop their knowledge, such as declarative and procedural knowledge along with decision-making (Weir et al., 2010). This provides greater motivation, persistence in the improvement of effort and confidence. When considering RAE, emotional development should also be taken into account. The player has a better self-concept (Thompson et al., 2004), as the effect for maturational advantage increases self-esteem.

Another item discussed has been the process of initial enrollment in soccer (Delorme et al., 2010) and hockey (Hancock et al., 2013), as RAE has been known to exist in non-competitive sporting contexts without prior selection (players less than seven years old). Delorme et al. (2010) discovered the existence of a phenomenon that prevented those born late in the year from starting to play soccer. It seems that parental influences are fundamental to the origin of this phenomenon, as some parents make their children participate in sports at quite early age, posing to them higher expectations of success and accelerating the processes of technical and tactic acquisition and development (Ward and Williams, 2003). Coaches must develop realistic expectations with regard to the physical abilities of younger players. These expectations should be based on biological characteristics and not chronological age (Deprez et al., 2013). Some of these explanations for the existence of RAE, like the self-concept, initial sports enrollment and family influences, seem to be logical, but require a greater empirical basis in order to be confirmed.

In fact, Baker et al. (2010) state that the maturational benefits in the early ages do not explain long-term sporting achievements, even though the maturational hypothesis is one of the main explanations experts have given for RAE. The whole and the interrelationships of these hypotheses can more fully justify this bias, as the environment and dynamics of players and soccer clubs are extremely complex to be considered from only one perspective. These theories may have a greater or lesser impact depending on the context.

The early streaming according to the level of expertise at an early age and the young players’ access to higher categories (Gutiérrez-Díaz et al., 2010) are factors that facilitate the increase of RAE. This is because they enable players with higher skills and experience to be trained by most qualified and experienced professionals (Sherar et al., 2007).

In the available literature, several possible solutions to reduce RAE are presented. Most authors have directed their proposals to changes in competitive systems (Gutiérrez-Díaz et al., 2010) or in the structure of youth participation in soccer to reduce the impact of RAE in the processes of talent identification and selection (Helsen et al., 2012):

1. To design calendars with alternative age limits of selection. For example, in 1995 in the USA, the date of selection was changed from the 1st of January to the 1st of July in an attempt to avoid RAE, but only the most favourable months towards the second half of the year were modified, as it was found similarly in European soccer (Helsen et al., 2000; Vaeyens et al., 2005).
2. To create smaller competition groups or with semiannual birth selection, not with annual or biannual periods, as it is usual in training competitions. This is more important to younger participants, as this is when the most influence of the maturational processes or changes in physical development exists.
3. To divide players into categories by the level of expertise, so that all players have the same opportunities. Delorme et al. (2010) suggest that the highest rates of sports dropouts occur among those who begin to play but find a factor of temporary inferiority in relation to physique compared with players born earlier in the year within the same category. Therefore, such solutions could be effective in amateur athletes who are less oriented to high performance.
4. To create categories by anthropometric characteristics: body mass and/or height. Setting a body mass or height limit within each competitive category would reduce the maturational effect. This solution seems more suitable for individual sports, especially opponent or fighting sports (González-Víllora and Pastor-Vicedo, 2012), but it seems hardly feasible in team sports.
5. To allow players born at the end of the year, and in which clearly a developmental and/or physiological difference is observed, to temporarily change to a youth age category.

These five solutions would be the easiest to carry out, but they have to be related to existing systems in soccer in each specific context or in the organisation of each country. However, at the same time, bureaucracy of organisational federations and authorities could be an impediment. Furthermore, there is no empirical evidence to conclude that one or more specific changes in competitions will definitely solve the RAE bias, although it is clear that projects of these changes to assess the effectiveness of the undertaken measures should be monitored, especially in the categories of training where the index of RAE is higher.

Other solutions to gradually resolve RAE might be to develop an optimal tryout system through small-sided games. In search of the solution, in order to reduce RAE in the USA, Horn and Okumura (2011) propose the change of the selection tests, where the destination of the players at short and medium term is decided. Tests should be designed to assess the technical and tactical quality of each player without giving priority to physical development, as coaches select the players based on physical attributes, which are more likely in relatively older athletes born in the same year (Hancock et al., 2013). Unnithana et al. (2012) state that success in soccer performance is the result of several interacting systems and that the approaches to identify sports talent should be holistic and based on multiple small-sided games. According to Horn and Okumura (2011), some game formats such as 8 vs. 8 should be eliminated and replaced with smaller ones (e.g., 3 vs. 3), allowing a greater opportunity for participation. Additionally, a distribution of clusters of competitions with young players should be made as small as possible: by quarters or semesters.

Promoters (politicians, associations and clubs) should include solutions aimed to progressively modify inner working of each sports organisation, so all professionals and family involved become aware that the highest performance of a player is given at the end of the training process, once the player has fully matured, i.e., in a long-term period. In some cases, more patience toward some sectors involved must be practised by not making an early specialisation or selection of early talent in order to reduce pressure on results in competitions.

The federations of different countries, UEFA and FIFA (Federation of International Football Associations) themselves, should become aware of the phenomenon of RAE and adapt the rules of competitions for the best development of all players on equal terms, regardless of their date of birth and other associated factors. With or without these necessary reforms, promoters, managers, coaches, physicians, psychologists and educators should train, supervise and be responsible for creating and developing equity for all those who want to be involved in sports. This will have a positive impact on the benefits and contribution of sports to future societies, as well as help develop better screening and training of sports talents, which will contribute to the enhanced performance of clubs and national teams.

The practical applications on RAE are as follows: 1) to analyse the effects of RAE in future international championships of all ages and genres, whether global or continental championships; 2) to assess RAE in regard to different relevant aspects that can be associated with greater or lesser intensity with this bias, which might be cognitive and physical aspects, tactical decisions, selection criteria and grouping, socio-cultural and economic influences, and other novel aspects; 3) to perform rule changes based on experience and to analyse their effects on the reduction or elimination of the bias resulting from RAE.

In conclusion, it can be stated that RAE was not evident in the professional category of the analysed selections, but it was in the three lower categories that were examined (youth categories), although the significance level and the effect size were greater in the U-21 and U-17 selections, this last selection being where the greatest evidence of the RAE was found. The teams that participated in the final stages of competitions were considered (quarter-final, semi-final and final), and it was observed that RAE had no evident impact on the professional category, however it influenced the lower categories, i.e., U-21, U-19 and U-17.

The presence of players born in the first half of their year of birth, among the teams which played in the quarter-finals, was also observed as in all the teams, excluding the Portugal U-19 selection, there was a greater number of players who were born in the first half of their year of birth rather than in the second. Therefore, the influence of the RAE still remains an unsolved issue in European football.

## Figures and Tables

**Table 1:** Birth distribution and effect size of professional, U-21, U-19 and U-17 soccer players

Category	Number and % of players per quarter					χ^2_3_^	*p*	Effect size *(d)*
	Q1 (%)	Q2 (%)	Q3 (%)	Q4 (%)	Total			
Professional	107 (29.1)	92 (25.0)	92 (25.0)	77 (20.9)	368	4.9	> 0.05	0.16
U-21	63 (34.2)	54 (29.3)	43 (23.4)	24 (13.1)	184	18.4	< 0.001	0.56**
U-19	53 (36.8)	35 (24.3)	30 (20.8)	26 (18.1)	144	11.8	< 0.01	0.45**
U-17	64 (44.1)	43 (29.7)	21 (14.5)	17 (11.7)	145	39.1	< 0.001	1.08**

* p < 0.05;

** p < 0.01.

**Table 2:** Birth distribution and effect size by category and team results

Category	Team Results	Number and % of players per quarter					χ^2_3_^	*p*	Effect size *(d)*

		Q1 (%)	Q2 (%)	Q3 (%)	Q4 (%)	Tota			
Professional	Quarters	54 (29.3)	44 (23.9)	44 (23.9)	42 (22.8)	184	1.913	> 0.05	0.13
	Semi-final	32 (34.8)	22 (23.9)	22 (23.9)	16 (17.4)	92	5.739	> 0.05	0.35
	Final	18 (39.1)	11 (23.9)	10 (21.7)	7 (15.2)	46	5.652	> 0.05	0.54
	Winner	10 (29.4)	4 (29.4)	5 (35.3)	4 (5.9)	23	4.304	> 0.05	0.44

U-21	Semi-final	28 (30.4)	35 (38.0)	17 (18.5)	12 (13.0)	92	14.174	< 0.01	0.80**
	Final	15 (32.6)	16 (34.8)	10 (21.7)	5 (10.9)	46	6.696	< 0.05	0.74*
	Winner	6 (26.1)	9 (39.1)	6 (26.1)	2 (8.7)	23	4.304	> 0.05	0.64

U-19	Semi-final	26 (36.1)	17 (23.6)	17 (23.6)	12 (16.7)	72	5.667	> 0.05	0.40
	Final	15 (41.7)	10 (27.8)	7 (19.4)	4 (11.1)	36	7.333	< 0.05	0.84*
	Winner	5 (27.8)	6 (33.3)	5 (27.8)	2 (11.1)	18	2.000	> 0.05	0.46

U-17	Semi-final	33 (45.8)	22 (30.6)	9 (12.5)	8 (11.1)	72	23.444	< 0.001	1.24**
	Final	15 (41.7)	9 (25.8)	8 (22.2)	4 (11.1)	36	6.889	< 0.05	0.71*
	Winner	7 (38.9)	4 (22.2)	5 (27.8)	2 (11.1)	18	2.889	> 0.05	0.46

Total	Semi-final	119 (36.3)	96 (29.3)	65 (19.8)	48 (14.6)	328	36.707	< 0.001	0.65**
	Final	63 (38.4)	46 (28.0)	35 (21.3)	20 (12.2)	164	24.049	< 0.001	0.70**
	Winner	28 (34.1)	23 (28.0)	21 (25.6)	10 (12.2)	82	8.439	< 0.05	0.50*

* p < 0.05;

** p < 0.01.
